# Tuberculosis of the breast with erythema nodosum: a case report

**DOI:** 10.1186/1752-1947-4-124

**Published:** 2010-04-29

**Authors:** Pao-Tsuan Kao, May-Yu Tu, Sai-Hung Tang, Hon-Kwong Ma

**Affiliations:** 1 Department of Internal Medicine, Cardinal Tien Hospital, Yongho Branch, Jhongsing Street, Yongho City, Taipei County, Taiwan 234; 2 Division of Infectious Disease, Cardinal Tien Hospital, Yongho Branch, Jhongsing Street, Yongho City, Taipei County, Taiwan 234; 3 Division of Chest Medicine, Cardinal Tien Hospital, Yongho Branch, Jhongsing Street, Yongho City, Taipei County, Taiwan 234; 4 Department of Radiology; Cardinal Tien Hospital, Yongho Branch, Jhongsing Street, Yongho City, Taipei County, Taiwan 234

## Abstract

**Introduction:**

There has been an increasing number of tuberculosis cases worldwide, but tuberculosis of the breast remains rare. In rare cases this is seen with a cutaneous manifestation of erythema nodosum.

**Case presentation:**

We report the case of a 33-year-old Chinese woman with tuberculosis of the left breast accompanied by erythema nodosum on the anterior aspect of both lower legs. Due to her poor clinical response to conventional therapy, and the histopathological findings of fine needle aspiration cytology, there were strong indications of tuberculosis. Her clinical diagnosis was confirmed by molecular detection of *Mycobacterium tuberculosis *complex by polymerase chain reaction. The diagnosis was further confirmed by a second polymerase chain reaction test of erythema nodosum which tested positive for *Mycobacterium tuberculosis* complex. She received anti-tuberculous therapy for 18 months, and finally underwent residual lumpectomy. During her follow-up examination after 12 months, no evidence of either residual or recurrent disease was present.

**Conclusion:**

Histopathological features and a high index of clinical suspicion are necessary to confirm a diagnosis of tuberculosis of the breast. Anti-tuberculous therapy with or without simple surgical intervention is the core treatment.

## Introduction

Tuberculosis (TB) is one of the leading infectious diseases worldwide. Extrapulmonary TB involving the breast is extremely rare. Clinical examination usually fails to differentiate breast TB from breast carcinoma. Vulnerability to breast TB is increased in women who are young, married, multiparous and who breast-feed [1]. Histopathological examination using fine needle aspiration cytology (FNAC) may reveal caseating epithelioid cell granulomas and acid-fast bacilli (AFB). Although the presence of an acid-fast stain or culture is essential to confirm diagnosis, it does not give a positive result in most patients [2,3]. Molecular detection of *Mycobacterium tuberculosis *by polymerase chain reaction can be particularly useful in the validation of a diagnosis of tuberculosis in clinical settings where the diagnosis is uncertain [3,4]. Anti-tuberculous chemotherapy is indicated for small lesions. In most cases, surgical intervention is reserved for persistent residual disease with severe disfiguration of the breast [3]. We report the first case of TB of the breast associated with a cutaneous manifestation of erythema nodosum.

## Case presentation

A 33-year-old Chinese woman was admitted to our surgical ward for fever with chills and a mass in the upper quadrant of her left breast. She had suffered from a left-sided mastitis that had been incised and drained at another institution 20 days prior to her presentation at our hospital. Poor wound healing with pus discharge was noted. She did not have any personal medical history of TB or diabetes mellitus. She also had no family history of breast cancer. She was married and had a three-year-old child.

Upon admission she had a body temperature of 38°C, blood pressure of 126/68 mmHg, a pulse rate of 89/minute, and a respiratory rate of 19/minute. On physical examination, we noted a firm mass of 5 × 6 cm with an erythematous open non-healing wound and a brownish discharge measuring 1.5 × 1.5 cm over the upper outer quadrant of her left breast. Dark reddish plaque skin lesions were found over both lower legs and the dorsal aspect of her feet. Her blood test results showed the following: white blood cells at 11.80 × 10^3^/μL, neutrophils at 77.3%, lymphocytes at 12.7%, platelets at 418 × 10^3^/μL, C-reactive protein at 4.9 mg/dL (normal range ≤ 0.8), and an erythrocyte sedimentation rate (ESR) during the first hour of 56 mm/hour (normal ≤ 12). Her blood culture revealed no growth, while her chest radiography was unremarkable.

**Figure 1:**
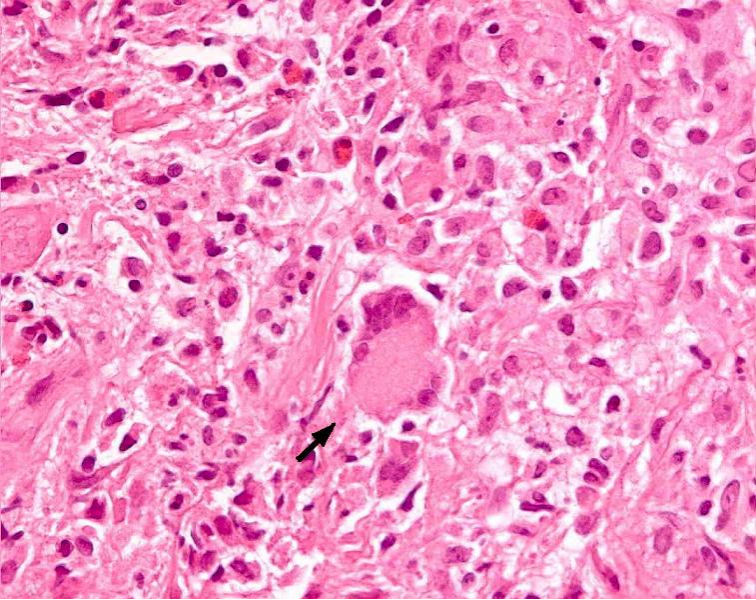
**Hematoxylin and eosin stain of our patient's breast tissue, magnification 400×, showing foreign body giant cell (arrow) and inflammatory cells**.

An ultrasonograph of our patient's left breast showed a lump measuring about 5 × 5 cm, which was conglomerated, with an irregular margin with hypoechoic heterogeneous echogenicity, and with a left axillary lymph node. An echo-guided core needle aspiration biopsy of her left breast was also performed which revealed a mastitis with granulation tissue. Under the microscope, this section of her left breast showed chronic mastitis mixed with granulation tissue and numerous foreign body giant cells but with no evidence of malignancy (Figure 1). A culture of the wound tissues failed to grow any organisms. Stains for AFB and TB culture were not undertaken. A dermatologist was consulted regarding the dark reddish plaque skin lesions, and a skin biopsy was later performed.

Her right lower leg skin biopsy showed granulomatous septal panniculitis that was consistent with erythema nodosum. Microscopically, there was fibrosis and granulomatous inflammatory cell infiltrate, primarily involving the thickened fibrous septa, but there was no evidence of vasculitis. A core needle biopsy tissue of her left breast was sent for a PCR test for *M. tuberculosis*. The result of the PCR test showed the presence of *M. tuberculosis *complex DNA. A right lower leg skin biopsy tissue was also tested for TB PCR and came back positive for *M. tuberculosis *complex DNA.

Our final diagnosis relied on histopathological tissue findings and on the molecular detection of *M. tuberculosis*. Our patient was then treated with anti-tuberculous medication after her PCR results were made available. After undergoing four months of anti-tuberculous treatment, her left breast mass was gradually reduced, but a new small mass appeared from the medial side of the initial mass. Excisional biopsy was done which revealed the presence of chronic granulomatous inflammation composed of epitheloid cells with Langhans giant cells, as well as small foci of necrosis. Although acid-fast stain and culture showed no tubercle bacilli, her anti-tuberculous therapy was continued. Her left breast mass gradually became smaller and then regressed. She was treated for 18 months without any further complication. After a further six months, she underwent lumpectomy. Her biopsy results revealed a fibroadenoma with a few foci of calcification of her breast tissue. She was regularly followed up for another 12 months and no evidence of the recurrence of her disease was noted.

## Discussion

Tuberculosis remains one of the leading causes of death from infectious diseases worldwide. Despite the fact that it can affect any organ or site of the body, the breasts, skeletal muscles and spleen are the most resistant to TB [5,6]. Tuberculosis comprises approximately 0.025% to 0.1% of all surgically treated diseases of the breast, but this ratio is higher in underdeveloped countries [7]. Although breast TB is primarily considered a disease of the developing world, a steady increase in the incidence of the disease has also been seen in developed countries. This is probably because of the migration of the infected population from endemic zones, and an increasing number of patients who are immunocompromised [8].

Tuberculosis usually occurs in women who are of a reproductive age. It is usually related to women who are breast-feeding and is extremely uncommon in older men [9]. Its clinical manifestations are variable. Constitutional symptoms such as fever, weight loss, night sweats, or a failing of general health are infrequently encountered [2]. Patients usually have a positive tuberculin skin test [10].

The common presentation of breast TB is a lump in the breast with or without ulceration associated with the sinus. Other presentations are diffuse nodularity and multiple sinuses. Multiple lumps are less common. Pain in the lump is present more frequently in breast TB cases than in breast carcinomas. The involvement of the nipple and the areola is rare in TB. Fixation of the skin, which resembles a neoplastic lesion, may also be present. Associated axillary lymphadenopathy is found in some patients [1,3,11]. Both breasts can be affected equally but bilateral involvement is very uncommon. Although the upper outer quadrant seems to be the most frequently involved site due to its proximity to the axillary nodes, any area of the breast can be affected [10].

Tuberculosis of the breast is mainly classified according to its primary and secondary forms. Cases that are of a primary form are quite rare. In its primary form, the only location of the disease is the breast. Infection spreads through a hematogenous or direct extension. Direct extension occurs when the infected material makes contact with the irritated skin or the breast ducts during lactation.

The secondary form of the disease occurs more frequently. When this happens, the patient usually has a prior history of TB. The main routes of spread are hematogenous, retrograde spread from the paratracheal, internal mammary or axillary group lymph nodes, or via a direct extension from the lung, pleural, mediastinum, costa, sternum and articular lesions [9,11,12]. In pregnant and lactating women, the breast is vascular with dilated ducts and is predisposed to trauma, thus making it more susceptible to TB infection [3]. Our patient's breast TB was presumed to be of a secondary form due to the presence of axillary lymph nodes.

Radiological imaging modalities like mammography and ultrasonography are unreliable in distinguishing breast TB from breast carcinoma. Similarly, computed tomography (CT) scan and MRI do not give a conclusive diagnosis without histopathological confirmation. CT scan is useful in differentiating between the primary and secondary forms. It is also helpful in evaluating the relationship between deeply located lesions with the chest wall and pleura and in detecting parenchymal lesions of the lung. As such it provides valuable guides to surgery and in defining the extent of the disease, including the involvement of the chest wall [11,12].

A correct diagnosis is confirmed by a combination of clinical suspicion and FNAC findings. Any form of breast TB may present with features of malignancy [6,11]. An accurate diagnosis is traditionally performed by demonstrating a classical caseation, AFB within such a lesion, and/or by demonstrating epitheloid granulomas, Langhans giant cells and lymphocyte aggregates. Although diagnosis is mainly based on the identification of tubercle bacilli, it has been recognized that an AFB-positive smear is not always sufficient evidence for a definitive diagnosis of *M. tuberculosis*. Differentiation of *M. tuberculosis *from other *Mycobacterium *species represents an important clinical evaluation [2]. Cultures and AFB staining are negative in most cases [3,4]. Failure to demonstrate necrosis on FNAC does not exclude TB because of the small quantity of the sample examined [3]. Open biopsy is still the most reliable test [1]. PCRs are highly sensitive especially in culture-negative specimens from paucibacillary forms of the disease and are necessary to distinguish it from other forms of granulomatous mastitis [3,4]. In our patient, PCR test of her left breast tissue showed the presence of *M. tuberculosis *complex DNA.

The cutaneous involvement of TB is rare. Underlying systemic involvement of TB is often seen in cutaneous TB, especially in children. Cutaneous TB is classified as true TB or tuberculids. True cutaneous TB is composed of tuberculous chancre, miliary TB, lupus vulgaris, scrofuloderma, TB verrucosa cutis, tuberculous metastatic abscess and orificial TB. Tuberculids are delayed sensitivity reactions to *M. tuberculosis *in patients with a strong immune response. Tuberculids include lichen scrofulosorum and papulonecrotic tuberculid. Facultative tuberculids consist of erythema induratum and erythema nodosum. Erythema induratum is a recurrent, painful subcutaneous nodule usually on the posterior aspect of the leg. Biopsy shows lobular panniculitis with vasculitis and granulomatous inflammation. Eythema nodosum is a painful subcutaneous nodule, mostly found on the anterior aspect of the leg. Biopsy shows septal panniculitis with an absence of vasculitis and usually without granuloma. Erythema nodosum often occurs in association with a granulomatous disease, including sarcoidosis, TB and granulomatous colitis. TB remains an important cause of erythema nodosum in endemic countries [13]. Our patient had developed erythema nodosum on the anterior aspect of both lower legs. PCR test on the erythema nodosum of her right lower leg also showed the presence of *M. tuberculosis *complex DNA.

Differential diagnosis most often includes carcinoma. Less common diseases are traumatic fat necrosis, plasma cell mastitis, chronic pyogenic abscess, mammary dysplasia, fibroadenoma, granulomatous mastitis, sarcoidosis, blastomycosis and actinomycosis [10,14]. Breast TB and breast carcinoma occasionally co-exist. It is important to remember that the recognition of TB does not exclude concomitant cancer [3,10].

Anti-tuberculous chemotherapy is still the main treatment for breast TB, and no specific guidelines are available for this kind of treatment. The disease should be treated as any other form of extrapulmonary TB. Anti-tuberculous therapy comprises rifampicin, isoniazid, pyrazinamide and ethambutol for the initial two months, which is then followed by rifampicin and isoniazid for another four months. The extension of anti-tuberculous therapy from 12 to 18 months is required in patients with slow clinical response, and complete resolution is obtained in most patients. FNAC should be repeated to confirm that the residual mass is fibrotic. In refractory cases that lead to breast destruction, a simple mastectomy may be performed [1,3,10,11]. The duration of follow-up after therapy is variable. In a study by Shinde *et al*., all patients were followed up for a minimum of two years to determine that they were free of the disease after therapy [1].

## Conclusion

In endemic TB regions, a painful breast mass with cutaneous manifestation of erythema nodosum is clinically relevant to determine a diagnosis of breast TB. Diagnosis is confirmed by histopathological findings, as well as molecular detection of *M. tuberculosis *using PCR. Anti-tuberculous chemotherapy is the core treatment, and minimal surgery is performed to remove any residual lesions.

## Abbreviations

AFB: acid-fast bacilli; CT: computed tomography; FNAC: fine needle aspiration cytology; PCR: polymerase chain reaction; TB: tuberculosis.

## Consent

Written informed consent was obtained from the patient for publication of this case report and any accompanying images. A copy of the written consent is available for review by the Editor-in-Chief of this journal.

## Competing interests

The authors declare that they have no competing interests.

## Authors' contributions

All authors contributed to all stages of this manuscript. All authors read and approved the final manuscript.
